# The Value of Success: Acquiring Gains, Avoiding Losses, and Simply Being Successful

**DOI:** 10.1371/journal.pone.0025307

**Published:** 2011-09-22

**Authors:** Samantha M. Mowrer, Andrew A. Jahn, Amir Abduljalil, William A. Cunningham

**Affiliations:** 1 Department of Psychology, The Ohio State University, Columbus, Ohio, United States of America; 2 Department of Psychological and Brain Sciences, University of Indiana, Bloomington, Indiana, United States of America; 3 Department of Radiology, The Ohio State University, Columbus, Ohio, United States of America; 4 Center for Cognitive and Behavioral Brain Imaging, The Ohio State University, Columbus, Ohio, United States of America; Nothwestern University, United States of America

## Abstract

A large network of spatially contiguous, yet anatomically distinct regions in medial frontal cortex is involved in reward processing. Although it is clear these regions play a role in critical aspects of reward-related learning and decision-making, the individual contributions of each component remains unclear. We explored dissociations in reward processing throughout several key regions in the reward system and aimed to clarify the nature of previously observed outcome-related activity in a portion of anterior medial orbitofrontal cortex (mOFC). Specifically, we tested whether activity in anterior mOFC was related to processing successful actions, such that this region would respond similarly to rewards with and without tangible benefits, or whether this region instead encoded only quantifiable outcome values (e.g., money). Participants performed a task where they encountered monetary gains and losses (and non-gains and non-losses) during fMRI scanning. Critically, in addition to the outcomes with monetary consequences, the task included trials that provided outcomes without tangible benefits (participants were simply told that they were correct or incorrect). We found that anterior mOFC responded to all successful outcomes regardless of whether they carried tangible benefits (monetary gains and non-losses) or not (controls). These results support the hypothesis that anterior mOFC processes rewards in terms of a common currency and is capable of providing reward-based signals for everything we value, whether it be primary or secondary rewards or simply a successful experience without objectively quantifiable benefits.

## Introduction

Areas of the striatum and medial frontal cortex (MFC) contribute to a neural network that facilitates reward-related learning and behavior. This extended network encompasses nucleus accumbens (NAcc) and spatially contiguous areas of medial prefrontal cortex (MPFC, approximately BA 10), medial orbitofrontal cortex (mOFC, medial BA 11), pregenual cingulate (pgACC, parts of BA 24 and 32), and subgenual cingulate (sgACC, parts of BA 24 and 25). Across studies, these regions have been shown to respond to a wide variety of reward types such as primary rewards[1], [2], secondary reinforcers such as money [3], [4], abstract points or money not actually paid out [5], [6], cognitive feedback [7], social rewards [8]–[10], and imagined events [11], [12], as well as rewarding non-events(or avoiding potential losses) [13].The current research attempts to further elucidate the subprocesses of this system by directly comparing outcomes that carry monetary consequences (tangible benefits) from others that simply indicate whether one was successful or not at a task (with no tangible benefits). Doing so allows us to decompose aspects of the reward system sensitive to the encoding of success from those sensitive to the updating of specific and quantifiable value representations.

According to component process models, the brain carries out multiple evaluative processes in decision-making, each with distinct but related functions [14]–[17]. Neuroimaging research has shown that different neural regions or different sub-parts of even the same region contribute to these processes. One crucial region involved in a host of these processes is medial orbitofrontal cortex (mOFC). However, a nuanced understanding of the processes carried out in the sub-parts of this heterogeneous brain region is in its early stage. One hypothesis regarding OFC function is that complexity of processing increases along an anterior/posterior gradient in mOFC, such that more posterior areas contribute to simpler processes or representations, whereas more anterior areas are capable of more complex and abstracted processes [18], [19]. According to this view, posterior areas may represent learned stimulus-value associations, whereas more anterior regions support more contextualized representations of value. Lending support to this perspective, Cunningham, Kesek, and Mowrer [5], demonstrated dissociations in stimulus- versus outcome-related processing within mOFC. In a decision task consisting of a series of gambles that participants accepted or rejected, a region of posterior OFC responded to stimulus values, or potential gains regardless of the outcomes that occurred, whereas a region of anterior OFC responded to outcome values, positive outcomes with respect to the choices participants made (both accepting gains and avoiding losses; see also Kim and colleagues [13], Wunderlich et al. [20]).

More abstracted representations of value allow for reward representations in a common currency [21]–[23], encoding not only tangible gains and losses but also more symbolic rewards such as social status and moral appropriateness. Medial OFC has been implicated in evaluating rewards along a common metric, similarly processing juice and monetary rewards [24], monetary and social rewards [25], and real and imagined rewards [26]. Chib, Rangel, Shimojo, and O'Doherty [27] showed that a potion of anterior mOFC responded similarly to the value of all types of goods (money, trinkets, and snacks) when participants engaged in a task in which they made purchasing decisions. Taken together, it appears that anterior mOFC may provide an abstracted currency signal. If so, then this region should provide a sense of value for all types of rewarding goods and also rewarding experiences. That is, anterior mOFC should process not only to outcome values that carry tangible benefits as previous research has shown, but also any successful outcome even without monetary or other tangible consequences.

For this investigation, we utilized functional magnetic resonance imaging (fMRI) to examine the processing of tangible gains and losses (monetary reward) to the cognitive representation of success and failure. Because in previous research both gains and non-losses were also by definition successes, we have not yet been able to disentangle whether particular neural regions activated to these rewards are processing successes more generally or are signaling an outcome with some tangible benefit that can be quantified. Our paradigm included non-monetary control stimuli in addition to monetary gains and losses, which allowed for a full comparison of different types of rewarding outcomes. Additional facets of the task (detailed below) allowed for random assignment to positive or negative outcomes, given an accurate response, in order to dissociate the outcome experienced from effort or performance. Thus, the current experiment had the capability of dissociating two possible roles for anterior mOFC – processing outcome value versus successful actions. We also explored other dissociations in reward processing by examining the possibility that other neural regions provided more nuanced evaluations of rewards.

## Methods

### Participants

Forty healthy, right-handed volunteers participated in this study (23 females; mean age 23.5, range 18–35 years). All participants gave written informed consent, and the study was approved by the Ohio State University Institutional Review Board for Biomedical Research. They reported no abnormal neurological history, and had normal or corrected-to-normal vision. A total of six individuals were excluded from further analyses. One participant was excluded due to failure to complete the experimental task, while another was an outlier showing brain activity differences greater than three standard deviations above the mean for all extracted regions (including critical areas such as prefrontal cortex and control regions such as those in visual cortices). Four additional participants were excluded due to computer failures in the experimental task. This left a total of 34 participants (18 females) for analysis. Compensation for participation independent of task performance was $35 in the fMRI study.

### Task

Participants encountered stimuli representing monetary gains and losses, which allowed for an examination of the similarities and differences in responses to receiving rewards (gains) and avoiding punishments (non-losses) both with tangible monetary benefits. Control stimuli had no monetary outcomes, but participants could respond to these correctly or incorrectly, thus providing the opportunity to experience the relatively more symbolic reward of being successful, unaccompanied by tangible, monetary reinforcement. Simple shapes (i.e., circles, squares, and triangles counterbalanced across participants) represented each stimulus type, which included gains, losses, and controls. Each stimulus required a specific button press and had two potential outcomes – success or failure. Successful outcomes included gains (+$1.00), non-losses (−$0.00), and correct responses to controls. Failures were non-gains (+$0.00), losses (−$1.00), and incorrect responses to controls.

**Figure 1:**
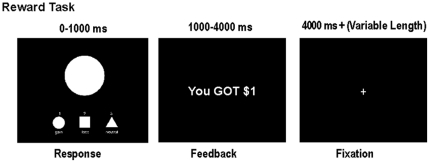
Example trial of the reward task. Stimuli were presented randomly for 1000ms. During this time, participants made a response by pressing a button corresponding to each stimulus type. A legend of the response options always appeared at the bottom of the screen. This was followed by 3000ms of feedback, which showed the outcome for each trial. Trials were separated by a fixation cross of variable length.

Each trial consisted of two phases: stimulus presentation with response lasting for 1000ms, and outcome feedback lasting for 3000 ms, followed by a variable length fixation cross (Figure 1). Stimuli were presented rapidly, and during the time a shape appeared on the screen, participants had to make a button press corresponding to each stimulus type. A legend of the response keys remained at the bottom of the display throughout. In order to dissociate outcome from performance, outcomes were determined randomly on the majority of trials. That is, for trials during which the correct response was made within 300–900 ms, there was a 50% chance of obtaining the successful outcome. To provide a feeling of control, incorrect responses and responses over 900 ms always led to the unsuccessful outcome; and correct responses under 300 ms always resulted in success. We told participants that they must respond within a “moving time window,” which they would be unaware of in order to make the task challenging in order to disguise the random nature of the majority of the outcomes. We also encouraged participants to respond first and foremost with accuracy since this was necessary for a positive outcome, and then with speed.

### Pilot Data

To examine emotional experiences elicited by this task and to determine that participants had emotional reactions even to success and failure trials in the control condition, a separate sample of 22 volunteers (14 females) participated in a pilot study outside of the scanner. Throughout the reward task, participants were asked to provide ratings of their emotions, thus allowing us to examine changes in subjective experience following the receipt of each type of outcome. Using a 1–5 scale anchored with labels “none” to “a lot,” participants rated the extent to which they felt the following emotions regarding the last outcome they experienced: joy/excitement, calm/relief, agitation/frustration, and dejection/disappointment. Emotions were presented in a random order each time the participants made ratings. During each run of the reward task, participants provided ratings at five different times – after four, five, six, seven, or eight consecutive trials of the reward task (determined randomly in the computerized task). In all, participants completed six runs of the reward task with 30 trials each (180 trials total) and five emotion ratings per run (30 ratings total). Compensation for participation independent of task performance was $8, and participants were given an additional $4 during the reward task. The latter amount of money was then affected by their task performance such that at the end of the study, we randomly selected four outcomes from the reward task that participants would actually receive. These outcomes were either added to or subtracted from the additional $4 participants were provided.

**Figure 2:**
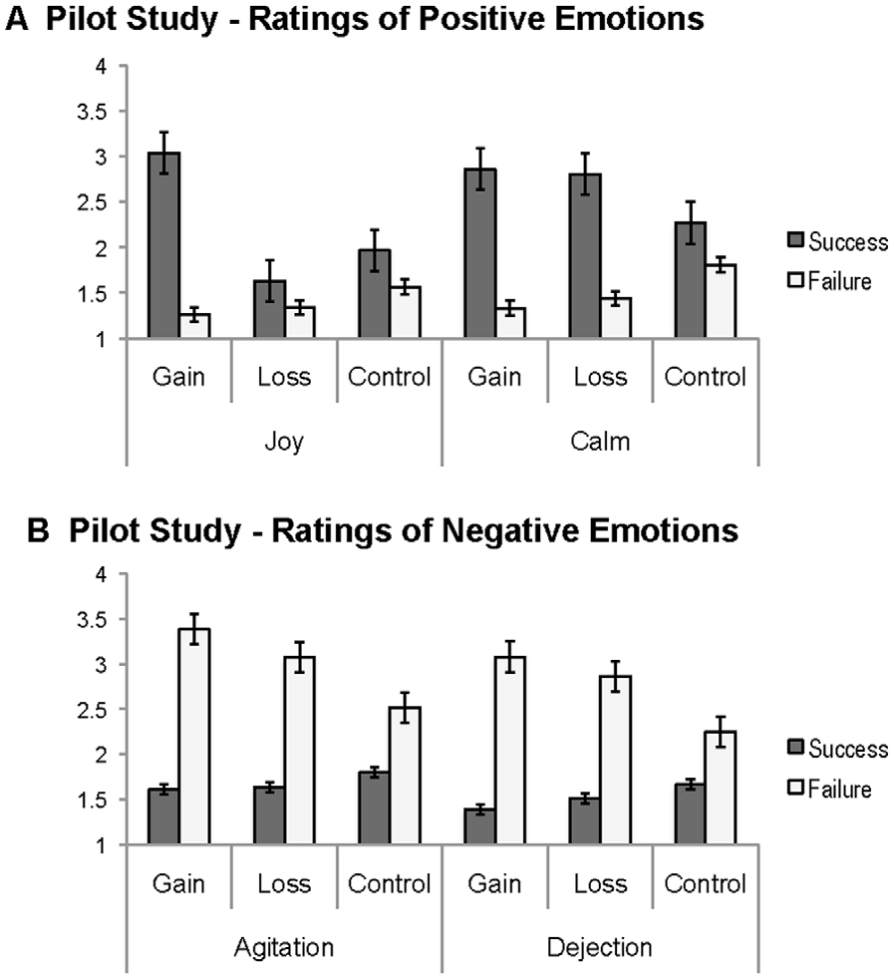
Pilot study results of emotion ratings during the reward task. Participants gave ratings of each of 4 emotions on a 1–5 scale for the last outcome they had experienced. Ratings occurred randomly every 4–8 trials and were presented in a random order for each type of emotion. Ratings of positive emotions joy/excitement and calm/relief by stimulus type and outcome (A). Ratings of negative emotions agitation/frustration and dejection/disappointment by stimulus type and outcome (B). Black bars represent standard errors.

In order to examine how the receipt of each type of outcome impacted participants' emotions, we conducted a MANOVA modeling the effects of stimulus (gain, loss, control), outcome (success, failure) and their interaction for each of the four rated emotions. We observed a significant interaction of outcome and emotion (*Wilk's λ* = .595, *F*_3,18_ = 4.08, *p*<.05) such that participants reported feeling more positive emotions following successes than failures (joy: *F*_1,5_ = 229.59, *p*<.01; calm: *F*_1,5_ = 217.99, *p*<.01) and more negative emotions following failures than successes (dejection: *F*_1,5_ = 219.17, *p*<.01; agitation: *F*_1,5_ = 247.23, *p*<.01). Further, participants reported more intense emotion ratings for gain and loss stimuli than controls as indicated by a main effect of stimulus type (*Wilk's λ* = .667, *F*_2,19_ = 4.74, *p*<.05). For ratings of joy, dejection, and agitation, contrasts showed that participants' ratings of these emotions were higher for gains and losses than controls (joy: *F*_1,5_ = 10.65; dejection: *F*_1,5_ = 8.53; agitation: *F*_1,5_ = 8.98; all *ps*<.01). However, there was no effect of stimulus type for ratings of calm (*p*>.50). Critically, for our experimental design, participants reported positive emotions for successes and negative emotions for failures even in control trials, as ratings of each emotion for control stimuli were significantly different from zero (all *ps*<.01). In other words, although these successes and failures did not result in monetary reward, participants reported appropriate (albeit weaker) emotional responses. These data indicate that our control condition successfully evoked emotional responses similar to monetary gains and losses despite no tangible rewards being provided on those trials. Interestingly, we also observed a main effect of emotion (*Wilk's λ* = .813, *F*_2,19_ = 5.31, *p*<.01), which showed a unique effect on ratings of joy such that the receipt of monetary gains impacted joy more than any other outcome, as joy ratings were significantly greater for successful gains than any other outcome (*F*_5,16_ =  63.71, *p*<.01). See Figure 2 for all pilot study results.

### fMRI Procedure

Using fMRI to examine the neural processing of different types of rewards, another group of participants completed the same reward task in the scanner with minor changes. First, we did not ask for emotion ratings so as not to interrupt the task. The task consisted of six runs of the reward task with 30 trials per run. Participants also completed 60 trials of the reward task to practice before entering the scanner. OptSeq [28] was used to generate optimized jittered trial sequences for efficient estimation of hemodynamic signal using lengths of 2000, 4000, 6000, or 8000 ms between trials. Overall, the average time between trials was 4000 ms (*SD* =  1847.24), and an equal number of each stimulus type was presented to participants such that they completed 60 trials each of gains, losses, and controls. In all, participants completed six functional runs of scanning, with each run lasting approximately five to six minutes. A ten second fixation cross appeared at the beginning and end of each run. For the scanning portion of the task in which real monetary outcomes occurred, participants were given an additional $15 and told that they could gain up to $30 or lose it all as a function of task performance (average total earnings  =  $14.47, range  =  $6 - $23). Instead of randomly selecting a subset of outcomes at the end of the experiment as was done for the pilot study, each trial affected their earnings. Participants saw their current earnings displayed at the end of each run and received their total earnings at the end of the experiment in addition to the $35 guaranteed for participation.

### fMRI Parameters and Processing

Imaging was conducted with a Philips 3T scanner. For whole-brain functional coverage, 40 axial slices (slice thickness  =  3.9 mm) were prescribed 20 degrees from the AC–PC line (see reference [29]). Nearly isotropic functional images were acquired from inferior to superior using a single-shot gradient echo planar pulse sequence (TE  =  22 ms, TR  =  2 s, in-plane resolution  =  3.4×3.4 mm, matrix size: 64×64×40, and FOV  =  220 mm). Prior to functional imaging, a high resolution T1 anatomical image (160 sagittal slices; 212 TE  =  3.75 ms, TR  =  25 ms; resolution  =  1.00°—0.43°—0.43 mm) was collected for normalization.

FMRI data were analyzed using SPM8 (Wellcome Department of Cognitive Neurology, London, UK; www.fil.ion.ucl.ac.uk/spm). During preprocessing, data were corrected for motion using SPM's Realign and Unwarp procedure. Then, each participant's EPI scans were co-registered to their corresponding T1 anatomical image, and unsegmented T1 images were spatially normalized to the SPM8 MNI template using the default settings. These transformations from the Co-registration and Normalisation procedures were then applied to the EPI functional images, and new images were interpolated to 3×3×3 mm dimensions. Functional images were then smoothed using a 6 mm FWHM (full-width-half-maximum) kernel.

### First-Level Analysis

Data were analyzed using the General Linear Model as implemented by SPM8. BOLD responses were estimated using a deconvolution analysis as a function of a canonical hemodynamic response function and its temporal derivative with a 160s high-pass filter. To generate estimates of neural activity associated with each stimulus type and outcome, time-series regressors were created from stimulus onset times for each combination of stimulus type, response accuracy, response latency, and outcome. Regressors of interest included trials during which participants responded both correctly and within 300–900 ms, yielding 6 crucial conditions produced by crossing the 3 stimulus types by 2 possible outcomes. Including only these 6 conditions provided a way to examine aspects of the experimental design that allowed for a dissociation of performance and outcome. Thus, neural activations could not be attributed entirely to perceived performance because incorrect responses were not included, and outcomes were randomly assigned for responses within 300–900 ms. Indeed, the majority of trials (89.78%) consisted of the 6 types of interest. All other condition combinations were modeled as regressors of no interest. There were 21 total combinations, however, not all of them occurred for each participant. Thus, regressors were modeled idiosyncratically for all participants.

## Results

In order to examine responses to different aspects of reward, we decomposed the effects of stimulus, outcome, and their interaction on neural activity. First level contrast images associated with each regressor of interest were subjected to a 3 (stimulus) x 2 (outcome) x subject ANOVA. Only the six crucial combinations of stimulus and outcome (i.e., where outcomes were randomly assigned) were included, thus allowing performance and task outcomes to be independent of each other. Using AlphaSim [30] to carry out Monte Carlo simulations to correct for multiple comparisons at an alpha threshold of *p*<.05 indicated that a cluster size of at least 18 contiguous voxels was required for correction. All reported effects survive this criterion unless otherwise noted.

### Main Effect of Outcome

**Figure 3:**
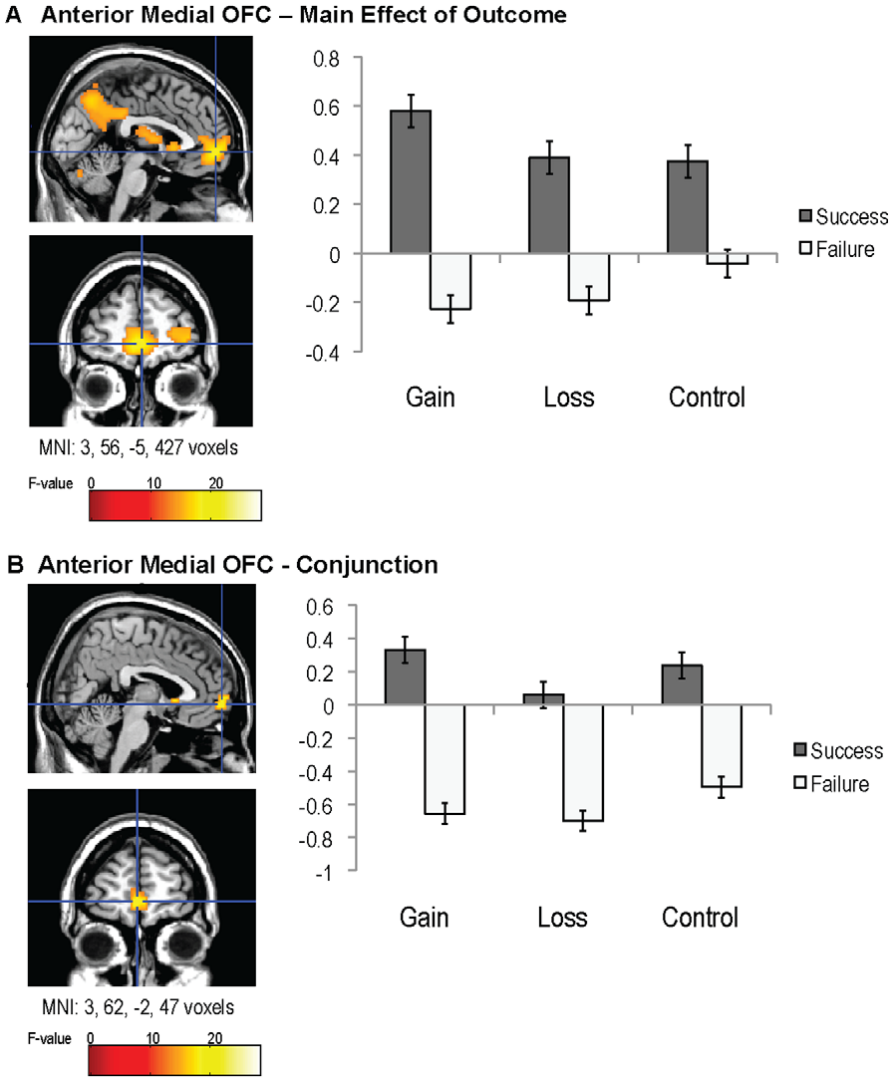
Outcome-related neural activations during the reward task in fMRI. A main effect of outcome was observed in anterior mOFC, such that this region responded to all successful outcomes regardless of stimulus type (A). A conjunction of successful gains, losses, and controls showed common success-related activity in a specific portion of anterior mOFC (B). The y-axis reflects parameter estimates (beta weights), and black bars represent standard errors.

Previous research has found outcome-related neural activity in anterior portions of mOFC such that this region responds to both monetary gains and non-losses. In this study, we asked if anterior mOFC is sensitive to any successful action, it should respond similarly to all positive outcomes, even correct controls. We found a main effect of outcome in an area of anterior mOFC (*F*_1,33_ = 45.39, *p*<.00001, MNI: 3, 56, −5, cluster size  =  427). Regardless of stimulus type, this region showed greater activity to successful outcomes than failures (Figure 3A). That is, anterior mOFC responded similarly to both monetary rewards (gains) and non-punishments (non-losses; cf. reference 12), as well as non-monetary rewards (correct controls). This region may provide a common metric for evaluating a range of rewarding outcomes, both concrete and relatively more abstract, or represent a feeling of “rightness” upon executing appropriate actions leading to good outcomes. Other regions showing a main effect of outcome and other effects are included in Table 1.

**Table 1:** Summary of neural activations observed for each F-test performed.

Effect	Anatomical Region (BA)	F-value	MNI Coordinates	Cluster Size
Outcome	Anterior Medial OFC (11/10)	45.39	3, 56, −5	2852
	Cerebellum	40.67	33, −64, −35	241
	Right Dorsolateral PFC (8)	36.36	27, 20, 58	325
	Inferior Parietal Lobe (7)	31.86	0, −64, 46	2828
	Right Mid Temporal Gyrus (21)	30.48	66, −7, −5	70
	Right Anterior MPFC (10)	21.69	27, 65, 7	25
	Right Inferior Temporal Gyrus (20)	20.26	57, −46, −14	53
	Right Putamen	19.05	27, 5, 4	34
	Right Fusiform Gyrus (37)	18.52	42, −61, −8	41
	Left Fusiform Gyrus (37)	18.06	−45, −58, −11	42
Stimulus	Right Inferior Parietal Lobe (40)	34.01	48, −43, 49	705
	Dorsal Anterior Cingulate (32)	32.36	6, 29, 37	1323
	Right Inferior Temporal Gyrus (20)	31.12	39, −19, −20	290
	Left Somatosensory Cortex (5)	27.98	−21, −49, 64	1269
	Right Visual Cortex, V3 (19)	25.12	33, −85, −2	190
	Left Visual Cortex, V2 (18)	21.31	−36, −91, −2	372
	Visual Cortex, V1 (17)	20.8	−9, −88, −2	35
	Left Inferior Parietal Lobe (7)	20.63	−30, −58, 43	148
	Right Inferior Frontal Cortex (44)	20.31	39, 8, 34	642
	Cerebellum	18.41	−9, −73, −29	102
	Left Insula (48)	17.2	−30, 17, −8	76
	Right Premotor Cortex (6)	15.85	27, −4, 55	61
	Right Somatosensory Cortex (2)	15.58	24, −43, 64	470
	Posterior OFC (11)	14.67	−21, 38, −14	51
	Left Angular Gyrus (39)	14.59	−48, −64, 22	134
	Left Posterior Insula (48)	14.12	−36, −16, 1	33
	Left Premotor Cortex (6)	11.52	−39, 2, 34	39
	Left Inferior Frontal Cortex (44)	11.17	−54, 23, 28	41
	Posterior Cingulate (23)	11.08	9, −4, 49	45
	Right Lateral OFC (11)	10.84	24, 62, 1	19
	Right Mid Insula (48)	10.19	42, −4, 10	18
	Right Mid Temporal Gyrus (21)	10.02	45, −55, 16	27
	Left Dorsal Anterior Cingulate (32)	8.89	−15, 41, 40	20
Interaction	Right Visual Cortex, V1 (17)	88.59	15, −82, 1	2577
	Pregenual Cingulate (24/32)	16.98	6, 47, 1	61
	Right Dorsolateral PFC (8)	12.46	27, 14, 55	59
	Right Angular Gyrus (39)	11.39	48, −67, 40	79

All activations meet the criteria of p<.001 and a cluster size of at least 18 contiguous voxels.

To provide additional support for the suggestion that this region of anterior OFC is associated with processing any type of success, even for non-monetary controls, we conducted a conjunction analysis to determine which regions were associated with greater activation for successes than failures for all stimulus types. This conjunction modeled the effects of successful gains(*T*_33_ = 6.46, *p*<.001), losses(*T*_33_ = 5.37, *p*<.001), and controls(*T*_33_ = 3.97, *p*<.001), and it revealed a distinct portion of OFC/MPFC that showed increased activation to all three types of rewards (*T*_33_ = 3.89, *p*<.001, MNI: 3, 62, −2, cluster size  =  47). Thus, OFC/MPFC provided a common representation of all reward types, both for concrete monetary rewards and more symbolic rewards that had no tangible consequences for participants (see Figure 3B).

### Interaction of Stimulus and Outcome

**Figure 4:**
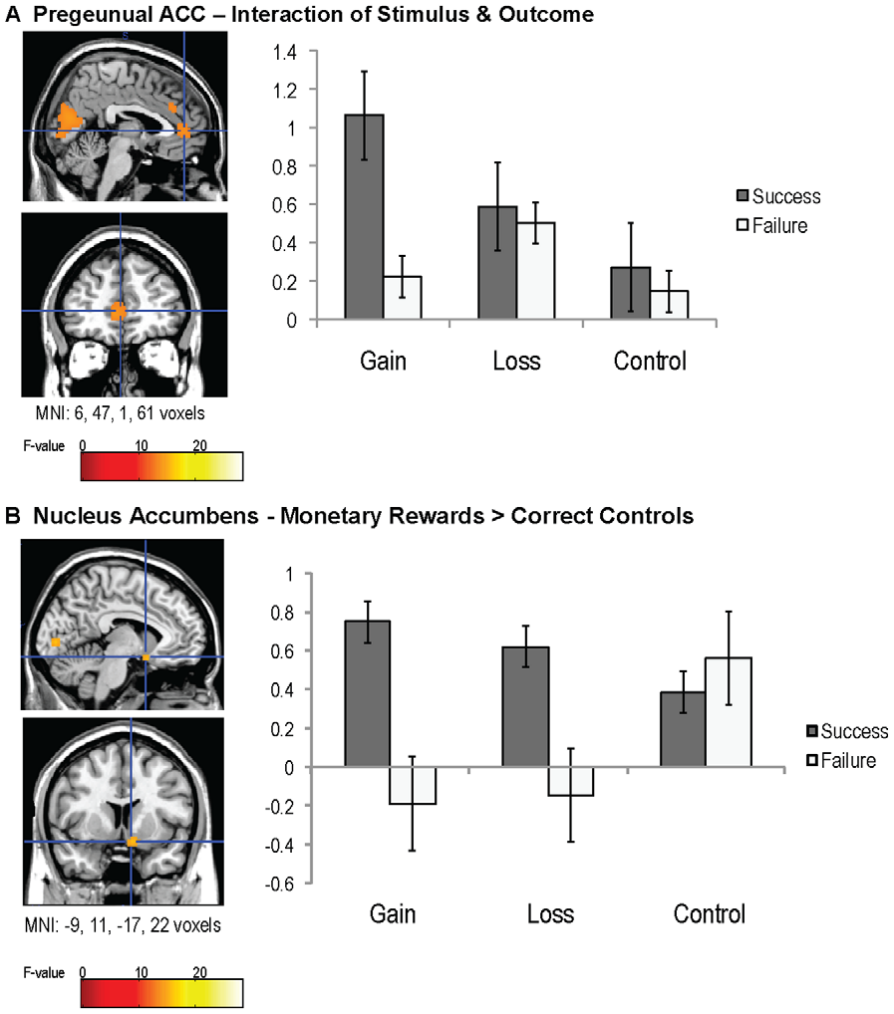
An interaction of stimulus and outcome was observed in two regions. Pregenual ACC specifically differentiated the reception of monetary gains (A), whereas NAcc differentiated only monetary successes, gains and non-losses, from failures (B). The y-axis reflects parameter estimates (beta weights), and black bars represent standard errors.

Although the processing of stimuli and outcomes can be considered independently, representations of outcomes can also be dependent on the type of stimulus encountered. That is, particular outcomes may be more or less rewarding depending on the stimulus type. Successfully obtaining gains may be processed and experienced differently from successfully avoiding a loss, or merely pressing the correct button. Thus, analysis of the stimulus-outcome interaction allowed for examination of neural regions that responded to rewards and punishments in a more nuanced, stimulus-dependent manner. We found that success-related activity in pregenual anterior cingulate (pgACC) was specific for gains (*F*_2,66_ = 16.98, *p*<.001, MNI: 6, 47, 1, cluster size  =  61). That is, this region showed the greatest activation when monetary gains were received (see Figure 4A). Interestingly, the pattern of neural activity found for this region mirrors the pattern of emotion results found in the pilot study for subjective reports of joy, an emotion that is linked to high arousal positive outcomes [31].

In addition to regions of medial frontal cortex, a planned contrast comparing monetary rewards (gains and non-losses) versus correct controls found that left nucleus accumbens showed activation to gains and non-losses (greater than non-gains and losses), but did not show any differences in the control trials (*F*_1,33_ = 17.31, *p*<.001 MNI: −9, 11, −17, cluster size  =  22; Figure 4B). A contrast comparing gains and loss trials to the control conditions indicated that unsuccessful monetary trials (non-gains and losses) showed less activation in NAcc than the two control trials (*t*_33_ = −4.24, *p*<.001) and that successful monetary trials (gains and non-losses) showed greater activation than the two control trials (*t*_33_ = 1.95, *p*<.05, one-tailed). This analysis suggests a potential dissociation between the reward processing in mOFC and in NAcc, with mOFC activation responding to a larger class of stimuli than the NAcc.

## Discussion

By understanding the events that lead to positive and negative outcomes, people can effectively direct their efforts and behaviors. Humans are capable of evaluating and comparing a wide range of rewards, Previous research has demonstrated that a variety of reward-related processes are carried out in areas of MFC. The current research extends this previous literature further by demonstrating that an anterior portion of mOFC plays a role in evaluating successful actions rather than simply processing tangible outcome values. Importantly, this finding links two hypotheses regarding OFC functions, common currency and abstraction, by showing that anterior mOFC contributes to abstracted currency processes.

The current research complements other evidence in support of the hypothesis that OFC provides a common currency whereby evaluations across stimulus modalities can occur. A common scale for value is necessary to navigate our complex environments that require a nearly constant stream of evaluations. Common currency allows for comparisons to be made between different types of rewards, and previous work has shown that mOFC processes the decision values for food, non-food, and money rewards similarly [27], for example. Our findings extend this to include the reward of being right and suggest that any type of successful action may be processed by anterior mOFC. This view of common currency suggests that even abstract and intangible rewards are processed similarly to tangible primary and secondary rewards. This ability to represent reward without immediate (or anticipated) tangible consequences may allow for the long-term development of feelings of competency, the honing of skills, and other processes in which one benefits from feeling successful and intrinsically motivated.

In addition to finding that anterior regions of OFC were associated with the processing of any type of success, we found evidence for other dissociations in process. In particular, we found that NAcc activity was only influenced by monetary successes, both gains and non-losses. Given this region's role in the prediction error signal [32], [33], it is likely that in our task, NAcc responded to outcome value, signaling tangible outcomes. Further, we observed that an area of pgACC was active when participants gained a dollar, and was relatively inactive for all other reward types. This pattern of means interestingly mirrored those in the pilot study for the subjective sense of joy participants rated. This joy specific relationship is consistent with findings that show that dysfunction in the pgACC is associated with reports of a lack of pleasure in response to rewards (anhedonia) or depressed mood [34], [35]. Thus, whereas some subdivisions of medial frontal cortex may be associated with any type of abstract success (and processing of non-loss information [13]), smaller subregions may represent only the more direct and concrete gain associated with receiving an extrinsic reward.

In sum, these data suggest that reward does not have a unitary representation, rather areas of the “reward system” provide multiple representations of reward.Although there is similarity in responding throughout the system (i.e., more activation for successes than failures), its constituents represent different types of reward-related information that can be used to guide learning and decision making in different ways. Importantly, this organization allows for a common scale along which vastly different rewards can be compared as well as flexibility in the system; thus, representations would be able to shift across individuals and situations.
